# Dual targeting of hepatic fibrosis and atherogenesis by icosabutate, an engineered eicosapentaenoic acid derivative

**DOI:** 10.1111/liv.14643

**Published:** 2020-10-28

**Authors:** Geurt Stokman, Anita M. van den Hoek, Ditte Denker Thorbekk, Elsbet J. Pieterman, Sanne Skovgård Veidal, Brittany Basta, Marta Iruarrizaga‐Lejarreta, José W. van der Hoorn, Lars Verschuren, Jimmy F. P. Berbée, Patrick C. N. Rensen, Tore Skjæret, Cristina Alonso, Michael Feigh, John J. P. Kastelein, Scott L. Friedman, Hans M. G. Princen, David A. Fraser

**Affiliations:** ^1^ TNO Metabolic Health Research Leiden The Netherlands; ^2^ Gubra Hørsholm Denmark; ^3^ Division of Liver Diseases Icahn School of Medicine at Mount Sinai New York NY USA; ^4^ OWL Metabolomics Parque Tecnológico de Bizkaia Zamudio Spain; ^5^ TNO Microbiology & Systems Biology Zeist The Netherlands; ^6^ Department. of Medicine Division of Endocrinology Einthoven Laboratory for Experimental Vascular Medicine Leiden University Medical Center Leiden The Netherlands; ^7^ NorthSea Therapeutics BV Amsterdam The Netherlands; ^8^ Department of Vascular Medicine Academic Medical Center University of Amsterdam Amsterdam The Netherlands

**Keywords:** apoptosis, arachidonic acid, atherosclerosis, lipotoxicity, NASH, oxidised phospholipids

## Abstract

**Background & Aims:**

While fibrosis stage predicts liver‐associated mortality, cardiovascular disease (CVD) is still the major overall cause of mortality in patients with NASH. Novel NASH drugs should thus ideally reduce both liver fibrosis and CVD. Icosabutate is a semi‐synthetic, liver‐targeted eicosapentaenoic acid (EPA) derivative in clinical development for NASH. The primary aims of the current studies were to establish both the anti‐fibrotic and anti‐atherogenic efficacy of icosabutate in conjunction with changes in lipotoxic and atherogenic lipids in liver and plasma respectively.

**Methods:**

The effects of icosabutate on fibrosis progression and lipotoxicity were investigated in amylin liver NASH (AMLN) diet (high fat, cholesterol and fructose) fed *ob/ob* mice with biopsy‐confirmed steatohepatitis and fibrosis and compared with the activity of obeticholic acid. APOE*3Leiden.CETP mice, a translational model for hyperlipidaemia and atherosclerosis, were used to evaluate the mechanisms underlying the lipid‐lowering effect of icosabutate and its effect on atherosclerosis.

**Results:**

In AMLN *ob/ob* mice, icosabutate significantly reduced hepatic fibrosis and myofibroblast content in association with downregulation of the arachidonic acid cascade and a reduction in both hepatic oxidised phospholipids and apoptosis. In APOE*3Leiden.CETP mice, icosabutate reduced plasma cholesterol and TAG levels via increased hepatic uptake, upregulated hepatic lipid metabolism and downregulated inflammation pathways, and effectively decreased atherosclerosis development.

**Conclusions:**

Icosabutate, a structurally engineered EPA derivative, effectively attenuates both hepatic fibrosis and atherogenesis and offers an attractive therapeutic approach to both liver‐ and CV‐related morbidity and mortality in NASH patients.

**Abbreviations**
AA: arachidonic acid
ALOX5AP: arachidonate 5‐lipoxygenase activating protein gene
ALT: alanine aminotransferase
AMLN: amylin liver NASH
AST: aspartate aminotransferase
CAT: catalase
CCR: chemokine receptor
Cer: ceramide
CETP: cholesteryl ester transfer protein
Col1a1: type 1 collagen α1
cPLA2: cytosolic phospholipase 2
CRP: C‐reactive protein
DAG: diacylglycerol
EPA: eicosapentaenoic acid
FXR: farnesoid X receptor
GSH: glutathione
GSSG: glutathione disulphide
H&E: haematoxylin and eosin
HETE: hydroxyeicosatetraenoic acid
HOMA‐IR: homoeostasis model assessment of insulin resistance
HYP: hydroxyproline
IL: interleukin
LA: linoleic acid
LDL‐R: LDL‐receptor
LPC: lysophosphatidylcholine
LPCAT: lysophosphatidylcholine acyltransferase
OCA: obeticholic acid
oxPL: oxidised phospholipid
PC: phosphatidylcholine
RPKM: reads per kilobase of transcript
SMA: smooth‐muscle actin
SOD: superoxide dismutase
SREBF: sterol regulatory element‐binding transcription factor
TAG: triacylglycerol
TGFRβ: transforming growth factor beta receptor
TGFβ: transforming growth factor beta
TNF‐: α; tumour necrosis factor alpha
TUNEL: terminal deoxynucleotidyl transferase dUTP nick end labelling
UHPLC‐MS: ultra‐high performance liquid chromatography mass spectrometry
VLDL: very low‐density lipoprotein

**Lay summary**

Liver scarring associated with obesity and diabetes rarely exists in isolation, and is typically part of a spectrum of disorders, including heart disease. Icosabutate is a novel treatment that, in mouse models, reduces both scarring of the liver and clogging of arteries. It is thus a promising therapy for subjects with both liver and heart disease.

## 1 INTRODUCTION

Although there is significant hepatic‐related morbidity and mortality associated with NASH, including cirrhosis, liver failure and hepatocellular carcinoma, the major overall cause of mortality in patients with NASH is cardiovascular disease (CVD), especially in patients who do not yet have advanced cirrhosis.^1^ Novel drugs for the treatment of NASH should thus ideally reduce both liver fibrosis and CVD, or at least avoid negative effects on CV‐risk factors. With respect to drugs currently being developed for the treatment of NASH, both beneficial^2^, ^3^ and adverse effects on plasma lipids^4^, ^5^, ^6^ and glycaemic control^7^ are reported.

Icosabutate is a liver‐targeted, semi‐synthetic, eicosapentaenoic acid (EPA) derivative under clinical development for NASH (NCT04052516). In addition to avoiding esterification via an α‐substitution that ensures portal vein uptake from the gut^8^ rather than peripheral distribution, an oxygen substitution in the β‐position limits its metabolism as a cellular energy source. The goal of the structural changes are to maximise hepatic concentrations of the free‐acid form for optimal targeting of both energy metabolism and inflammation via omega‐3 fatty acid responsive pathways, for example, nuclear and G‐protein coupled receptors^9^, ^10^ and the arachidonic acid (AA) cascade.^11^ Several lines of evidence, including the recently reported beneficial effect of aspirin in NASH patients,^12^ suggest AA metabolism is involved in the progression of liver fibrosis.^13^, ^14^, ^15^ The potential of icosabutate to target both energy metabolism and inflammation is suggested by its ability to rapidly reduce both plasma lipids and, of particular relevance to NASH, elevated liver enzymes in hyperlipidaemic subjects.^16^, ^17^, ^18^

We recently reported that icosabutate improved early hepatic fibrosis and inflammation in a prevention design NASH rodent model.^8^ However, as studies in humans are targeting established NASH, proof of efficacy in a delayed‐treatment study design are more relevant. Additionally, the anti‐fibrotic effects of icosabutate were compared with rosiglitazone, which has not demonstrated improvements in fibrosis in humans.^19^ We have therefore evaluated the dose response effects of delayed treatment with icosabutate in an established biopsy‐confirmed AMLN *ob/ob* mouse model of NASH^20^ and compared its activity to a farnesoid X receptor (FXR) agonist, obeticholic acid (OCA), which has demonstrated benefits on liver histology in humans.^21^ We have complemented these findings by assessing effects of icosabutate directly on hepatic stellate cells, the key fibrogenic cell in liver.^22^

As rodents transport plasma cholesterol primarily in the HDL fraction and are resistant to atherogenesis, transgenic models with human‐like lipoprotein metabolism were used. To this end, mice expressing the human ApoE3‐Leiden (APOE*3Leiden) isoform and human ApoC1 cross‐bred with human Cholesteryl Ester Transfer Protein (CETP) transgenic mice were utilised to study treatment effects on hyperlipidaemia and atherosclerosis. The APOE*3Leiden.CETP mouse is a well‐established model with a human‐like response to all lipid‐modulating interventions that are being used in the clinic.^23^, ^24^

## 2 MATERIALS AND METHODS

### 2.1 Animals, treatments and analyses

#### 2.1.1 AMLN *ob/ob* mouse model of NASH

Animal experiments were conducted according to internationally accepted principles for the care and use of laboratory animals (licence no. 2017‐15‐0201‐01378, The Animal Experiments Inspectorate, Denmark). The animal protocol was designed to minimise pain or discomfort to the animals. Male B6.V‐Lepob/JRj *(ob/ob*) mice, 5‐week‐old at the arrival, were obtained from Janvier Labs (Le Genest Saint Isle, France) and housed in a controlled environment (12 hour light/dark cycle, light on at 3 am, 21 ± 2°C, humidity 50 ± 10%). Each animal was identified by an implantable microchip (PetID Microchip, E‐vet, Haderslev, Denmark). Mice had ad libitum access to tap water and a diet high in fat (40%, containing 18% trans‐fat), 40% carbohydrates (20% fructose) and 2% cholesterol (AMLN diet; D09100301, Research Diets, New Brunswick, NJ).

A total of 60 male *ob/ob* mice were fed the AMLN diet for 18 weeks then, after biopsy‐histological confirmation of steatosis and fibrosis, randomised into 5 ob/ob‐NASH groups of 12 mice to receive either 45, 90 or 135 mg/kg bw/d (mpk) icosabutate *per os* (PO), 30 mpk OCA acid (PO) or no treatment (control) for a further 8 weeks. All animals included in the experiments underwent pretreatment liver biopsy, as described in detail previously.^25^ Only mice with fibrosis stage ≥1 and steatosis score ≥2, evaluated using the clinical criteria outlined by Kleiner et al^26^ were included in this study. As control, one group continued the regular AMLN diet. Body weight was measured daily and food intake twice weekly during the treatment period.

As supportive mechanistic evidence for effects observed in the AMLN *ob/ob* model, hepatic expression of relevant target genes are presented from a screening study in male *ob/ob* mice fed the AMLN diet for 15 weeks then randomised (without biopsy) to treatment with either icosabutate 112 mpk PO or vehicle control for 4 weeks (8 mice per group).

#### 2.1.2 APOE*3Leiden.CETP transgenic mouse model

All APOE*3Leiden.CETP mice were housed and bred at the animal facility of The Netherlands Organization for Applied Scientific Research (TNO). All experimental procedures were approved by the Animal Care and Use Committee of the respective institute. To study (a) the effects of icosabutate on plasma lipids and lipoprotein metabolism, and (b) atherosclerotic plaque formation, APOE*3Leiden.CETP transgenic mice (C57BL/6J background) received a semi‐synthetic cholesterol‐rich Western‐type diet (WTD) containing 0.15% (females) or 0.25% (males) (w/w) cholesterol. After a 4‐week run‐in period, mice were randomised based on plasma total cholesterol (TC) and triacylglycerol (TAG), body weight and age. (a) To study the effect of icosabutate on plasma lipids and lipoprotein metabolism, 7‐10‐week‐old male APOE*3Leiden.CETP transgenic mice (n = 8 per group) received WTD supplemented with 112 mpk icosabutate, 30 mpk fenofibrate^27^, ^28^ or vehicle control for 4 weeks. (b) To examine the effect on atherosclerotic plaque formation, 11‐15‐week‐old female APOE*3Leiden.CETP mice (n = 15 per group) received WTD, WTD containing 37.5 mpk (first 4.5 weeks of treatment) or 15 mpk (final 12.5 weeks of treatment) icosabutate or WTD with 30 mpk fenofibrate for an additional 17 weeks. All animals received food and water ad libitum. All authors had access to the study data and had reviewed and approved the final manuscript.

See Supplementary Methods and Data for more details of in vivo and in vitro materials and methods.

### 2.2 Statistical analyses

Analyses were carried out by the respective institutions performing the studies. For the AMLN *ob/ob* model a two‐way ANOVA with Tukey's multiple comparisons test was performed for body weight and quantitative histological analyses. A one‐way ANOVA with Dunnett's post‐hoc test was used for all other parameters except hepatic gene expression for which a two‐tailed Student's *t* test was used (GraphPad Prism v7.03 software). For the APOE*3L.CETP studies statistical differences between groups were determined by using non‐parametric Kruskal‐Wallis followed by Mann‐Whitney U test for independent samples (SPSS software). For LX‐2 cell studies one‐way ANOVA with Dunnett's post‐hoc test was performed (GraphPad Prism v7.03 software). For hepatic lipidomics, differences between groups were tested using Student's *t* test (MassLynx 4.1 software). For all studies a *P* < .05 (≤.05 for APOE*3L.CETP studies) was considered statistically significant and all results are shown as mean ± standard error of mean (SEM).

## 3 RESULTS

### 3.1 Both icosabutate and OCA reduce hepatic steatosis, but only icosabutate reduces plasma ALT and hepatic macrophage numbers in AMLN *ob/ob* mice

**FIGURE 1:**
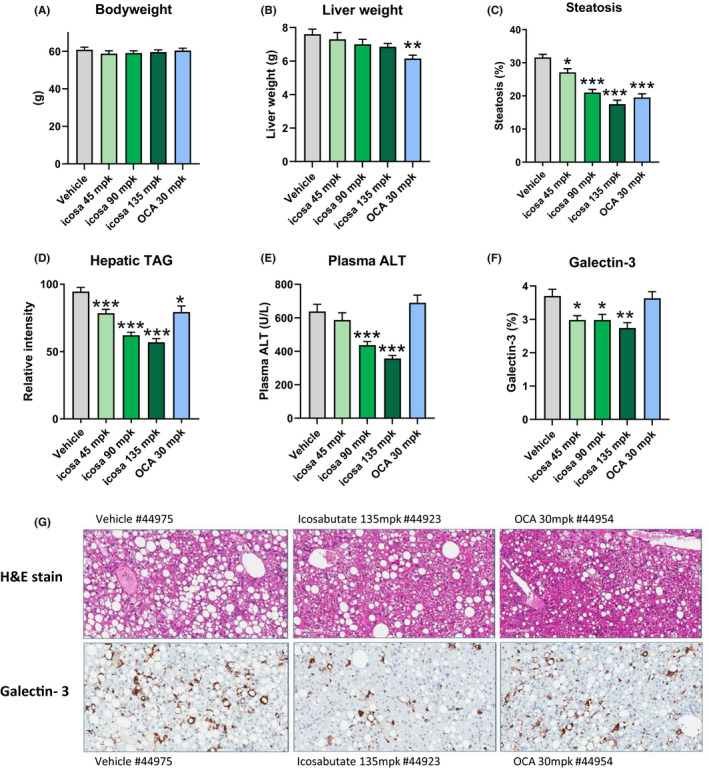
Both icosabutate and OCA reduce liver fat, but only icosabutate reduces liver enzymes and liver inflammation in AMLN *ob/ob* mice. Effects of treatment on terminal bodyweight (A), liver weight (B) and liver lipids as measured by % steatosis (C) or hepatic triacylglycerol content (D), plasma ALT (E) and hepatic galectin‐3 (F). Values represent mean ± SEM for 12 mice per group. (G) Representative histological photomicrographs of liver cross sections stained with H&E or anti‐Galectin 3, magnification 20x. *P < .05, **P < .01, ***P < .001 vs vehicle

In the AMLN *ob/ob* model neither icosabutate nor OCA affected bodyweight (Figure 1A). OCA significantly reduced liver weight by 19% (*P* < .05) at 8 weeks whereas icosabutate had no significant effect (Figure 1B). A dose‐dependent decrease in steatosis (Figure 1C) in response to icosabutate treatment was seen, with the 135 mpk dose achieving a 47% reduction while OCA reduced liver fat by 38% (both *P* < .001). Icosabutate also elicited a dose‐dependent effect on hepatic TAG lowering (Figure 1D), with reductions of 17, 35 and 40% (all *P* < .001), while OCA achieved a reduction of 16% (*P* < .05).

With respect to liver injury and inflammation, only icosabutate (90 and 135 mpk) reduced plasma ALT (−32 and −44% respectively, *P* < .001) (Figure 1E). To further assess the effects of either treatment on hepatic inflammatory macrophage infiltration, quantitative immunohistochemistry was performed to assess hepatic galectin‐3 (Gal‐3) content. All doses of icosabutate achieved a significant reduction in hepatic Gal‐3 (Figure 1F). Representative histological photomicrographs of liver cross sections stained with H&E and galectin‐3 are shown in Figure 1G. The decreases in Gal‐3 in response to icosabutate treatment occurred in conjunction with significant decreases in mRNA transcripts for key genes (eg TNF‐α, TGF‐β1, TGFRβ and CCR2) regulating hepatic inflammatory responses in AMLN *ob/ob* mice after 4 weeks treatment with icosabutate (FigureS1, section D).

### 3.2 Icosabutate prevents progression of hepatic fibrosis in AMLN *ob/ob* mice

**FIGURE 2:**
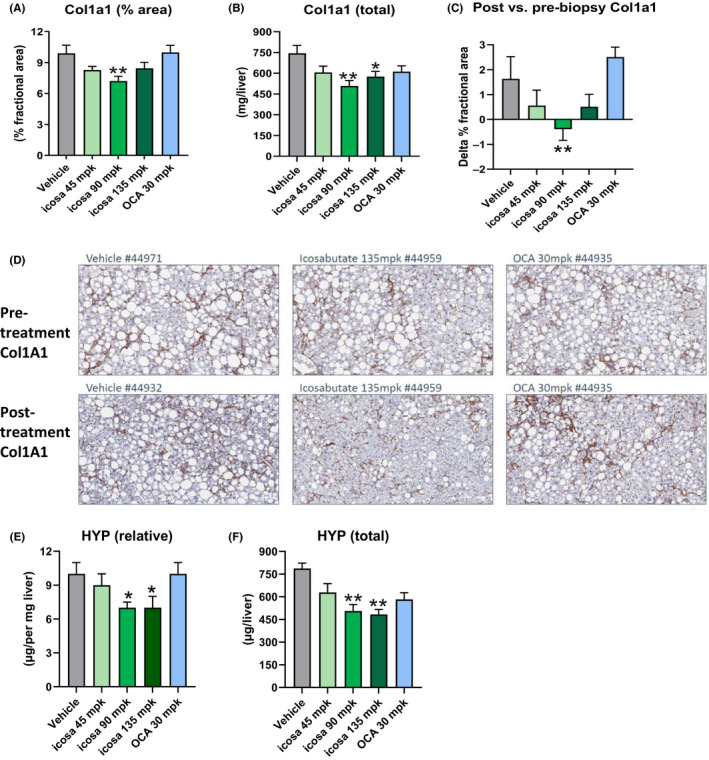
Icosabutate prevents hepatic collagen deposition in AMLN *ob/ob* mice. Liver col1A1 content as measured by either percent area (A) or total content (B). Change in liver col1A1 content between baseline and post‐treatment (C). Histological photomicrographs of liver cross sections stained with anti‐col1A1 pre‐ vs post‐treatment (D), magnification 20×. Liver hydroxyproline (HYP) content (E‐F). Values represent mean ± SEM for 12 mice per group. *P < .05, **P < .01, ***P < .001 vs vehicle

To assess the effects of treatment with either icosabutate or OCA on fibrosis, hepatic concentrations of hydroxyproline (HYP) were measured via a biochemical assay in addition to type 1 collagen α1 (col1A1) protein content measured via quantitative immunohistochemistry. Icosabutate reduced hepatic col1A1 content expressed as % area at the 90 mpk dose by 27% (*P* < .01, Figure 2A) and as total col1A1 at both the 90 and 135 mpk doses (−32%, *P* < .01 and −23%, *P* < .05 respectively, Figure 2B). The study design employed a prebiopsy confirmation of fibrosis as an inclusion criterion. Change in col1A1 content (post‐biopsy minus prebiopsy, Figure 2C) demonstrates that all doses of icosabutate minimised the progression of fibrosis during the treatment period, albeit only the 90 mpk dose was significant (*P* < .01) vs vehicle. Representative histological photomicrographs of liver cross sections stained for col1A1 pre‐ or post‐treatment are shown in Figure 2D.

Only icosabutate (at the 90 and 135 mpk doses) significantly reduced HYP content expressed in both relative and total units with both the lowest icosabutate dose and OCA having no significant effect (Figure 2E and F respectively). In summary, the combined col1A1 and HYP data demonstrate that icosabutate effectively inhibits the progression of fibrosis.

The decreases in hepatic fibrosis in response to icosabutate treatment occurred in conjunction with significant decreases in mRNA transcripts for multiple genes regulating stellate cell activation, fibrogenesis and fibrolysis in AMLN ob/ob mice after 4 weeks treatment with icosabutate (Figure S1, section A).

### 3.3 Icosabutate reduces hepatic myofibroblast content in AMLN *ob/ob* mice in vivo and proliferative responses of human stellate (LX‐2) cells in vitro

**FIGURE 3:**
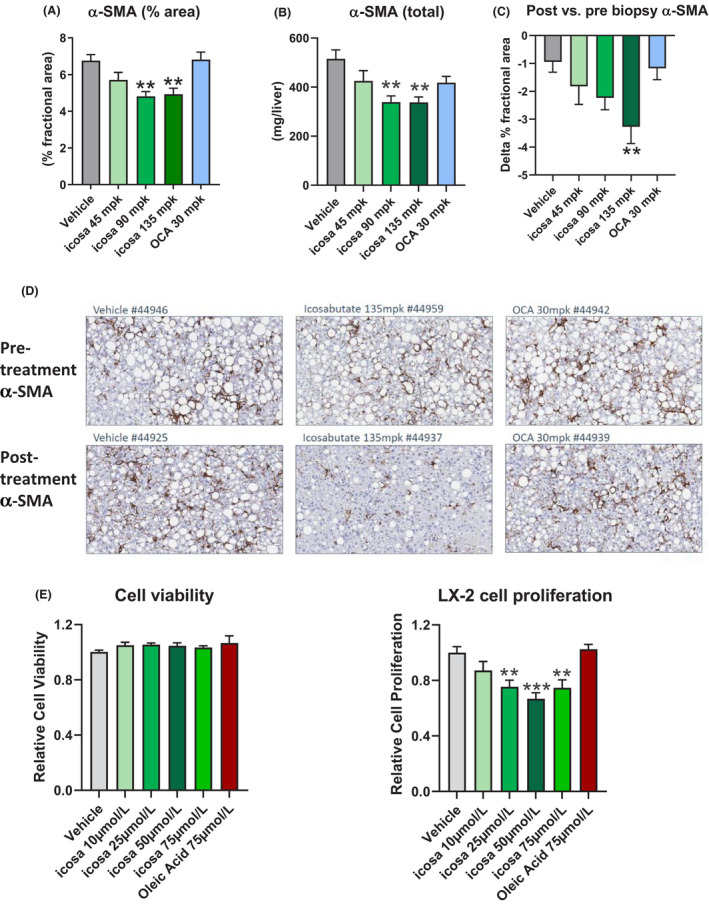
Icosabutate reduces the hepatic content of activated stellate cells in AMLN *ob/ob* mice and proliferation of human stellate cells. Liver α‐SMA content as measured by percent area (A) and total content (B). Change in liver α‐SMA content between baseline and post‐treatment (C). Histological photomicrographs of liver cross sections stained with anti‐α‐SMA (a marker of activated stellate cells) pre‐ vs post‐treatment (D), magnification 20×. Values represent mean ± SEM for 12 mice per group. *P < .05, **P < .01, ***P < .001 vs vehicle. LX‐2 cell viability (E) and proliferative responses (F). Results are presented as normalised mean values ± SEM of 5 (2 for OA) independent experiments performed in triplicate. **P < .005, ***P < .0001 vs vehicle

To gain further insight into underlying drivers of the decrease in fibrosis with icosabutate treatment, α‐SMA content was measured as a marker of myofibroblast content. Icosabutate 90 and 135 mpk reduced α‐SMA content expressed both as % area and total (all *P* < .01, Figure 3A and B respectively) whereas OCA and icosabutate 45 mpk had no significant effect. Icosabutate 135 mpk also led to a significant reduction in post‐ vs prebiopsy α‐SMA content (Figure 3C). Representative histological photomicrographs of liver cross sections stained with α‐SMA pre‐ or post‐treatment are shown in Figure 3D. Overall, α‐SMA data demonstrate that icosabutate prevents the development of fibrosis in association with a decline in myofibroblast numbers.

In association with the finding of decreased myofibroblast content in response to treatment with icosabutate, we performed additional in vitro experiments in spontaneously proliferating LX‐2 cells, a widely used human hepatic stellate cell line. As fatty acids can serve as a fuel for proliferating stellate cells via autophagy,^29^ oleic acid (75 μmol/L) was included as a control. While no change in cell viability was observed at any dose, a significant reduction in LX‐2 cell proliferation was observed at the 25, 50 and 75 μmol/L icosabutate doses with the strongest inhibition (−33%, *P* < .001) seen at the 50 μmol/L dose (Figure 3E). These data suggest that the decrease in myofibroblast content in vivo may be driven by direct anti‐proliferative effects of icosabutate on myofibroblasts.

### 3.4 Icosabutate reduces hepatic lipotoxicity and downregulates the hepatic arachidonic cascade in AMLN *ob/ob* mice

**FIGURE 4:**
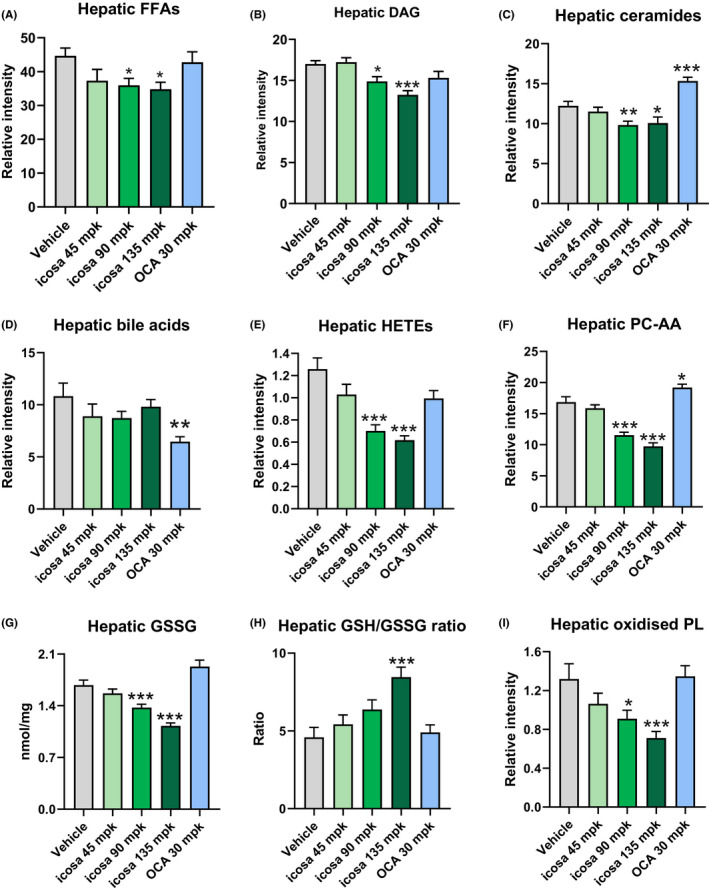
Icosabutate reduces hepatic NASH‐associated lipotoxic lipids species, oxidative stress and oxPLs in AMLN *ob/ob* mice. Post‐treatment hepatic concentrations of FFAs (A), DAG (B), ceramides (C), bile acids (D) HETEs (comprising 11(R)‐, 12‐ and 15(S) isomers) (E), AA‐containing PC (F), oxidised glutathione (G), reduced/oxidised glutathione ratio (H) and oxPLs (comprising PC‐AA‐OH and LPC‐LA‐OH) (I). Values represent mean ± SEM for 12 mice per group. *P < .05, **P < .01, ***P < .001 vs vehicle

In addition to lowering hepatic TAG, icosabutate (at 90 and 135 mpk) significantly decreased hepatic free fatty acids (FFAs, Figure 4A), diacylglycerols (DAG, Figure 4B) and ceramides (Figure 4C) whereas OCA significantly increased ceramides and reduced bile acids (Figure 4D) only.

Icosabutate (90 and 135 mpk) reduced HETEs (total 5‐, 11[R]‐ and 15[S]‐HETEs) by 45 and 51% respectively (*P* < .001) whereas both the lowest dose of icosabutate and OCA were without significant effect (Figure 4E). To assess if the marked decrease in HETEs could be mediated via decreased AA stores, concentrations of AA in the dominant membrane phospholipid, phosphatidylcholine (PC), were measured. As shown in Figure 4F icosabutate induced a marked and significant decrease in PC‐AA at the 90 mpk (−32%, *P* < .001) and 135 mpk (−42%, *P* < .001) doses while a small significant increase was seen in response to OCA treatment.

Expression of genes regulating hepatic HETE formation demonstrated a significant decrease in ALOX5AP (regulating 5‐HETE and LTB4 generation) mRNA transcripts after treatment with icosabutate. Transcripts for the lipoxygenase genes, ALOX5 and ALOX15, were reduced but constitutive expression levels were low (Figure S1, section B). With respect to PC‐AA content, a significant decrease was also observed in LPCAT2 expression, which is responsible for incorporation of AA into cellular membranes, along with a trend towards lower expression of cPLA2, responsible for AA release. Taken as a whole, the data suggest a downregulation of the AA metabolism by icosabutate at multiple junctures, including decreased AA stores in both PC and DAG stores, decreased HETE concentrations and downregulation of genes regulating eicosanoid synthesis and AA membrane remodelling.

### 3.5 Icosabutate reduces hepatic oxidative stress and oxidised phospholipids in AMLN *ob/ob* mice

As highly unsaturated fatty acids are susceptible to peroxidation and can increase hepatic oxidative stress,^30^ we also measured hepatic oxidised (GSSG) glutathione and the GSSG/reduced glutathione (GSH) ratio as a marker of oxidative stress and cellular redox status.^31^ Icosabutate (90 and 135 mpk) significantly decreased hepatic GSSG concentrations (Figure 4G), which in turn was largely responsible for the markedly increased GSH/GSSG ratio (84% increase, *P* < .01) seen with the highest icosabutate dose (Figure 4H). In conjunction with the increased GSH/GSSH ratio, significant decreases in the hepatic expression of genes regulating enzymatic antioxidants catalase (CAT), and superoxide dismutase 1 and 2 were also observed (Figure S1, section C).

With respect to the functional consequence of increased hepatic oxidative stress, it has recently been demonstrated that hepatic oxidised phospholipids (oxPL) are a causal driver of NASH.^32^ We therefore also measured a mixture of the dominant oxPLs (PC 16:0/AA‐OH and LPC 16:0/LA‐OH) and found that icosabutate significantly reduced their concentration by 31% (*P* < .05) and 46% (*P* < .001) for the 90 and 135 mpk doses respectively, whereas no effects were seen in response to OCA treatment (Figure 4I).

### 3.6 Icosabutate primarily targets highly unsaturated DAG and TAG species in AMLN *ob/ob* mice

**FIGURE 5:**
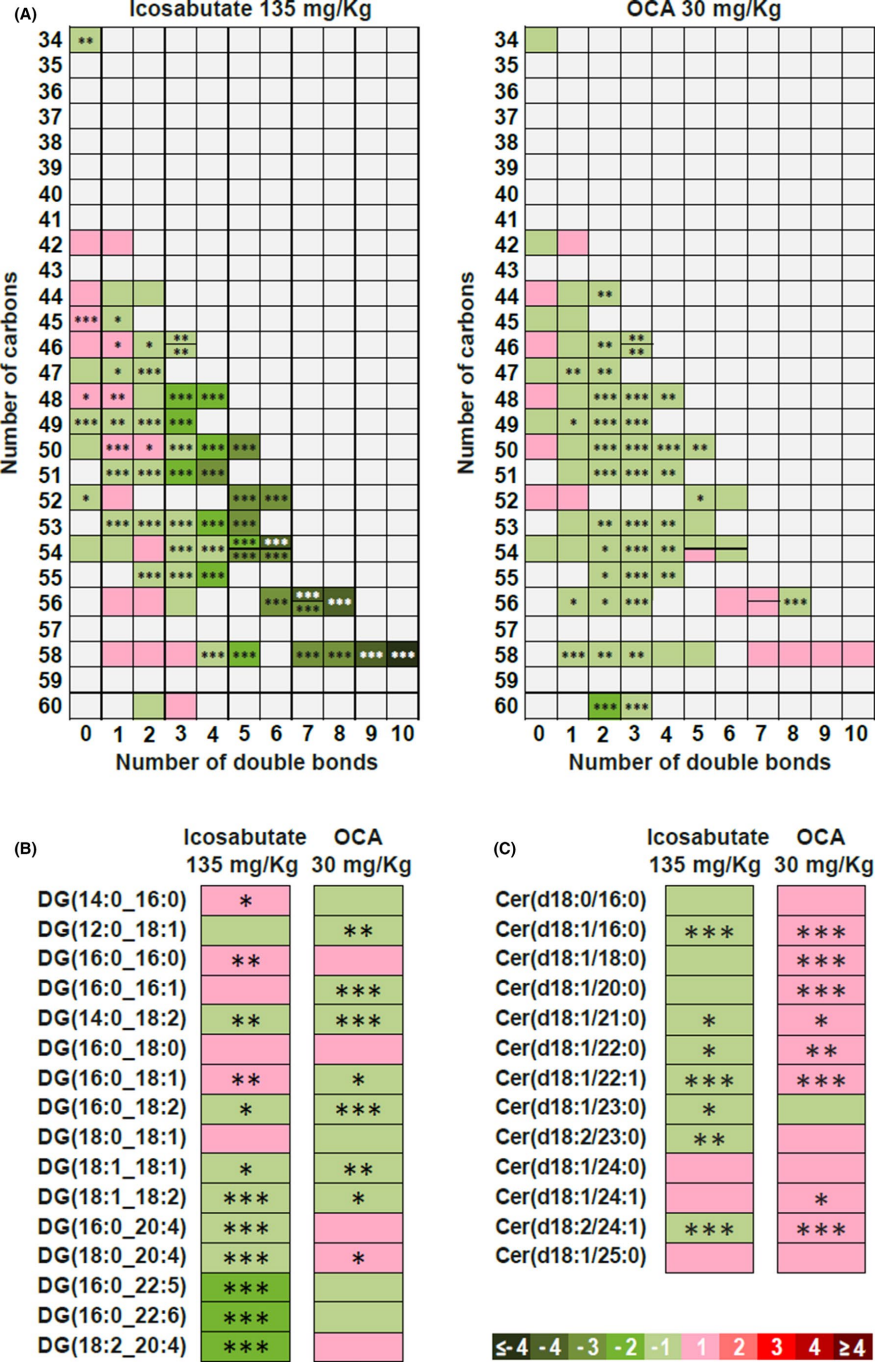
Icosabutate preferentially targets highly unsaturated hepatic TAG, DAG and reduces ceramides in AMLN *ob/ob* mice. Influence of the number of carbons and double bond content in the decrement of TAG (A). Change in specific DAG (B) and ceramide (C) species. Colour code represents the transformed ratio between means of the groups: green sections denote metabolites that were reduced (negative log_2_ fold‐changes) and red sections denote increased metabolites (positive log_2_ fold‐changes). For TAG the y axis denotes the number of carbons and the x axis the number of double bonds. Data are presented as mean ± SEM, *P < .05, **P < .01, ***P < .001 vs vehicle

As specific DAG and ceramide species are associated with steatohepatitis^30^ and insulin resistance^31^ in addition to serving as the main source of hepatic AA,^32^ we also analysed qualitative differences within the main lipid classes in response to icosabutate (135 mpk shown) or OCA. Whereas OCA primarily reduced TAG and DAG species with 2 to 4 double bonds, icosabutate induced a remarkable reduction in the long and highly unsaturated TAG and DAG species, including AA containing species (Figure 5A and B). Changes in hepatic ceramide species demonstrated a different pattern (Figure 5C), with reductions in response to icosabutate in both shorter and longer chain species, including a 30% reduction in Cer(d18:1/16:0), a species with a deleterious effect on glycemic control.^31^ Overall the data suggest major differences between icosabutate and OCA with respect to both quantitative and qualitative changes in hepatic lipotoxic lipid species.

### 3.7 Icosabutate reduces hepatic apoptosis in AMLN *ob/ob* mice

**FIGURE 6:**
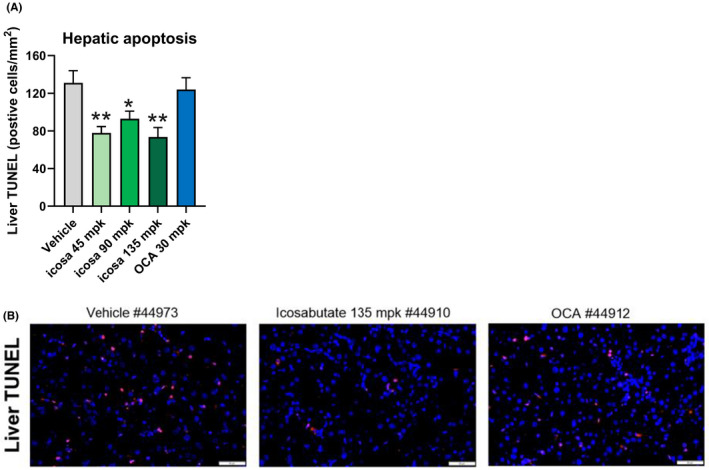
Icosabutate reduces hepatic apoptosis in AMLN *ob/ob* mice. Effects of treatments on the number of apoptotic cells numbers at study termination as measured by TUNEL (A). Representative images of cross sections of liver stained with TUNEL at termination (magnification 20×, scale bar = 50 μm) (B). Values represent mean ± SEM for 12 mice per group. *P < .05, **P < .01 vs vehicle

As a decrease in apoptosis could provide a mechanistic link between the treatment related changes in hepatic lipotoxicity/oxidative stress and the decrease in fibrosis,^1^ we performed a terminal deoxynucleotidyl transferase dUTP nick end labelling (TUNEL) assay in all groups at study end. Icosabutate reduced apoptotic cell numbers by 41% (*P* < .01), 29% (*P* < .05) and 44% (*P* < .01) vs vehicle in the 45, 90 and 135 mpk dose groups respectively (Figure 6A). In contrast, OCA had no significant effect on apoptotic cell numbers. Representative images of liver cross sections stained with TUNEL are shown in Figure 6B. Overall the data demonstrate that icosabutate effectively decreases apoptosis in AMLN *ob/ob* mice.

### 3.8 Icosabutate decreases plasma TAG and total cholesterol but does not affect HDL cholesterol in APOE*3Leiden.CETP mice

**FIGURE 7:**
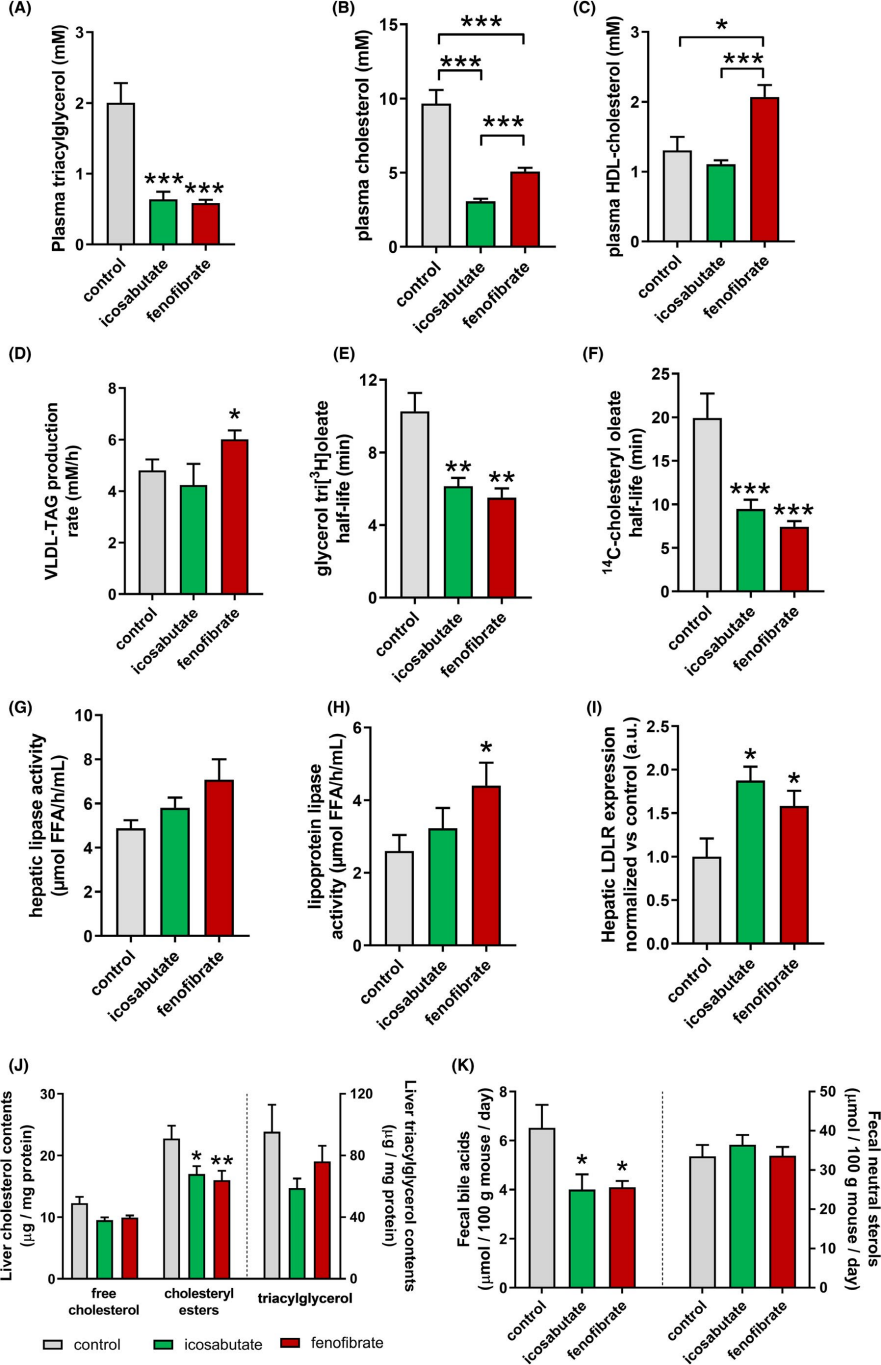
Icosabutate reduces plasma lipids in association with increased hepatic clearance and upregulated hepatic LDL‐R expression in APOE*3Leiden.CETP mice. Total plasma TAG (A), plasma cholesterol (B) and HDL cholesterol (C). (D) Hepatic VLDL‐TAG production expressed as production rate per hour (mM/h). (E) Hepatic VLDL clearance was determined by measuring glycerol tri[^3^H]oleate‐derived activity and (F) [^14^C]cholesteryl oleate in plasma and expressed as plasma half‐life. Activity of (G) hepatic lipase and (H) lipoprotein lipase was determined in post‐heparin plasma as the rate of free fatty acid (FFA) release from VLDL‐TAG. (I) Protein expression of LDL‐R in liver tissue was determined by Western blotting. Data were normalised vs controls. (J) Liver free cholesterol, cholesteryl esters and triacylglycerol contents. (K) Excretion of bile acids and neutral sterols in faecal samples. Data are presented as mean ± SEM, *P ≤ .05, **P ≤ .01, ***P ≤ .001

The effects of 4 weeks of treatment with either icosabutate or fenofibrate on the main circulating plasma lipids were assessed in APOE*3Leiden.CETP mice. Both icosabutate and fenofibrate significantly decreased plasma TAG (Figure 7A) to a similar extent (−70% for both compounds, *P* < .001), whereas icosabutate had a more pronounced effect on total cholesterol (−68% and −47% respectively, both *P* < .001, Figure 7B). Fenofibrate increased HDL cholesterol by 62% (*P* < .05) whereas icosabutate was without effect (Figure 7C).

### 3.9 Icosabutate increases VLDL plasma clearance and hepatic uptake of cholesterol and TAG in APOE*3Leiden.CETP mice

To investigate the mechanism/s by which icosabutate lowers plasma lipids, we evaluated very low‐density lipoprotein (VLDL) production and clearance as determinants of plasma VLDL‐TAG levels as compared to fenofibrate.^28^ In contrast to icosabutate, fenofibrate increased VLDL‐TAG production compared to controls (+25%) (Figure 7D). Since VLDL‐associated ApoB production and VLDL composition were similar in both groups (data not shown) these data indicate production of larger TAG‐rich VLDL by fenofibrate as reported previously.^28^

Compared to controls, the half‐life of glycerol tri[^3^H]oleate (−40%, *P* = .008; −46%, *P* = .005, respectively) (Figure 7E) and [^14^C]cholesteryl oleate (−52%, *P* = .001; −63%, *P* = .001, respectively) (Figure 7F) were decreased to a similar extent in both icosabutate‐ and fenofibrate‐treated mice. Total uptake of glycerol tri[^3^H]oleate‐derived lipids was most prominent in liver and skeletal muscle tissue, whereby icosabutate and fenofibrate increased ^3^H‐activity in the liver compared to vehicle controls (Figure S2A). Uptake of [^14^C]cholesteryl oleate by the liver was increased by both treatments compared to controls (Figure S2B). Post‐heparin activity of hepatic lipase (Figure 7G) and lipoprotein lipase (Figure 7H) did not significantly differ between control‐ and icosabutate‐treated animals, whereas fenofibrate increased lipoprotein lipase activity as reported previously.^28^ Combined, these data show that the lipid‐lowering effect of icosabutate is mediated through increased hepatic VLDL remnant clearance and may be independent from lipase‐mediated hydrolysis of VLDL‐TAG.

LDL‐R‐mediated hepatic uptake of ApoB‐containing lipoproteins is the most important pathway for removal of these atherogenic particles from the circulation. Both icosabutate and fenofibrate increased hepatic LDL‐R protein expression compared to controls (Figure 7I). These findings indicate that icosabutate increases LDL‐R‐mediated cholesterol uptake in the liver.

### 3.10 Effect of icosabutate on liver lipid content and faecal bile acid excretion in APOE*3Leiden.CETP mice

To assess if reductions in plasma lipids were associated with hepatic cholesterol/TAG accumulation, decreased cholesterol absorption or increased excretion, hepatic lipid concentrations of cholesterol and TAG along with faecal excretion of bile/neutral sterols were measured. Both treatments decreased hepatic cholesteryl ester content (Figure 7J). Compared to controls, faecal excretion of bile acids was reduced by icosabutate and fenofibrate, whereas neutral sterol contents were unaffected (Figure 7K). These findings negate the possibility that liver accumulation accounts for the decrease in plasma lipids.

### 3.11 Icosabutate modulates hepatic expression of genes regulating lipid metabolism and inflammation in APOE*3Leiden.CETP mice

A partial overlap between icosabutate and fenofibrate was observed for differentially expressed genes (DEG) (Figure S3A), which could be expected given that fatty acids and their metabolites act as endogenous ligands for PPAR‐α.^9^ In line with the physiological data, upregulated DEG in the icosabutate group were enriched for pathways involved in lipid metabolism, including fatty acid metabolism and β‐oxidation, and (chole)sterol biosynthetic processes (Figure S3B). Downregulated pathways include complement activation, the AA cascade, innate immune response and acute phase response‐related pathways (Figure S3C). Using computational pathway analysis, Z factor scores were calculated to identify the most dominant transcription factors involved in transcriptional control following icosabutate treatment. Transcription factors that are predicted to be involved in icosabutate‐mediated pathway activation are PPAR‐α and ‐γ, and sterol regulatory element‐binding transcription factor (SREBF)‐1 and −2 and PPAR gamma coactivator (PGC)‐1α (Figure S3D).

### 3.12 Prolonged low‐dose icosabutate decreases atherosclerotic lesion formation in APOE*3Leiden.CETPmice

Given the marked effect of icosabutate treatment on reduction of plasma cholesterol and TAG, we examined if icosabutate affects aortic root plaque formation in APOE*3Leiden.CETP mice. For 17 weeks animals were treated with vehicle, low‐dose (37.5 mpk for first 4.5 weeks of treatment and 15 mpk for 12.5 weeks) icosabutate, or fenofibrate. This dose regimen achieved a reduction in cholesterol exposure that approximated the 29% reduction in LDL‐C observed in hypercholesterolaemic subjects after treatment with 600 mg/day icosabutate for 28 days.^33^

**FIGURE 8:**
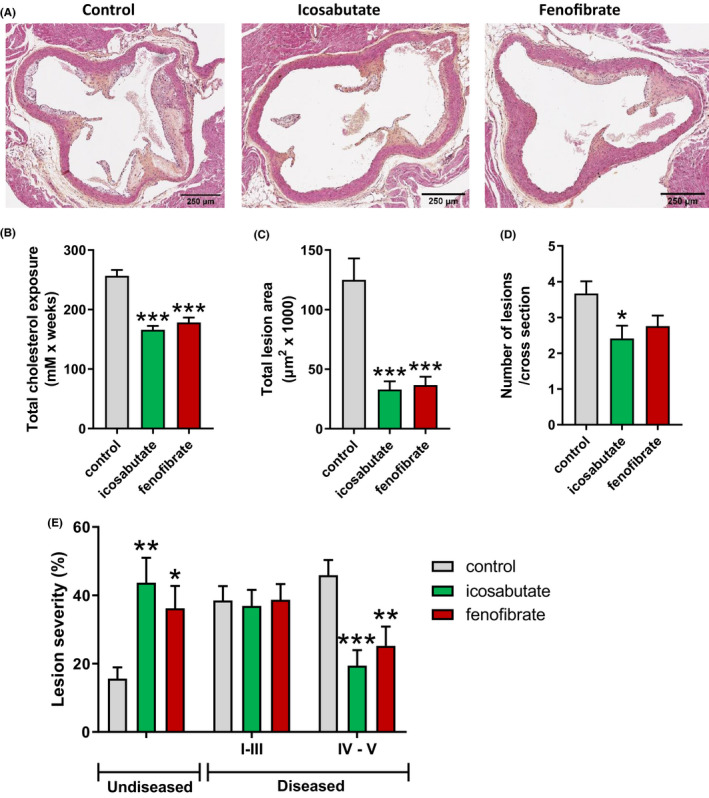
Icosabutate reduces atherosclerotic plaque formation in APOE*3Leiden.CETP mice. Representative images of the aortic root (A). (B) Total cholesterol exposure. (C) Total lesion area per cross section of the aortic root. (D) Number of lesions per cross section. (E) Percentage of undiseased aortic root segments and pathohistological scoring of lesions expressed as percentage of total number of lesions. Severity was classified as type I‐II: initial lesion/fatty streak, type III: intermediate lesion, type IV‐V: (fibro)atheroma lesion. Data are presented as mean ± SEM, *P ≤ .05, **P ≤ .01, ***P ≤ .001

Representative histological photomicrographs (Figure 8A) demonstrate reduced plaque formation and smaller plaques in the icosabutate‐ and fenofibrate‐treated mice. Reductions in total cholesterol exposure were comparable for the icosabutate and fenofibrate groups and significantly lower than the control group (Figure 8B). Total lesion area was similarly reduced by both icosabutate and fenofibrate (Figure 8C) whereas only icosabutate significantly reduced lesion number (Figure 8D). Both compounds significantly reduced the formation of severe type IV‐V lesions with a concomitant increase in undiseased segments (Figure 8E) albeit these changes were more pronounced in the icosabutate‐treated group. These findings show that low‐dose icosabutate treatment effectively decreases both the development and severity of atherosclerotic lesions.

## 4 DISCUSSION

Utilising a delayed treatment model of established AMLN diet‐induced NASH,^20^, ^25^ the current findings demonstrate that icosabutate effectively targets NASH‐associated hepatic fibrosis. The anti‐fibrotic effects compared favourably with OCA, a compound with demonstrated benefits on liver histology in humans.^21^ The improvement in fibrosis occurred in conjunction with a significant decrease in myofibroblast content, the key driver of fibrogenesis in the liver.^22^ Furthermore, novel data demonstrate attenuation of atherogenesis by icosabutate in APOE*3Leiden.CETP mice in association with increased hepatic LDL‐R protein expression and a more rapid hepatic uptake of plasma TAG and cholesterol.

Mechanistically, the anti‐fibrotic effect of icosabutate observed in AMLN *ob/ob* mice was associated with reduced hepatic αSMA content, a marker of myofibroblasts. This was reflected not only by comparison to control *ob/ob* mice at study end (cross sectional) but also in comparison to the pretreatment biopsies in the icosabutate group (temporal). There are several possible and potentially overlapping explanations for this observation, including decreased transformation of stellate cells into proliferating myofibroblasts, reverting myofibroblasts to a quiescent state and/or inducing cell death. The decrease in fibrosis in response to icosabutate is a likely consequence of the decreased myofibroblast content and was accompanied by a significant reduction in the expression of genes regulating both fibrogenesis (β‐PDGFR, PDGF‐ β, COL1A1) and fibrolysis (HSP‐47, LOXL2, TIMP1). The decrease in proliferation of LX‐2 cells supports a direct anti‐proliferative effect of icosabutate. The mechanism underlying this effect requires further evaluation, but as human stellate cells minimally express PPAR‐α and show no changes in proliferative responses in response to incubation with PPAR‐α agonists, the mechanism is likely PPAR‐α independent.^34^

Decreases in specific hepatic ceramides also provide a mechanistic link not only to NASH but also to atherogenesis and insulin resistance.^35^ In particular, the 30% decrease in Cer(d18:1/16:0), a species with established negative effects on glycemic control,^31^ may contribute to the significant improvement in glycaemic control observed in hypertriglyceridaemic subjects.^17^ Conversely, the significant increase in hepatic ceramides [including Cer(d18:1/16:0)] seen in response to treatment with OCA in AMLN *ob/ob* mice may explain the worsening of HOMA‐IR observed in humans in response to OCA.^7^ The significant decrease in hepatic DAG, with established negative effects on glycaemic control,^36^ may also contribute to the aforementioned beneficial effects of icosabutate.

Activation of inflammatory pathways is of well‐established relevance to both NASH^37^ and CVD.^38^ The anti‐inflammatory effects of icosabutate observed in the AMLN *ob/ob* model are substantiated by the hepatic transcriptomic data from the APOE*3Leiden.CETP model that demonstrates broad inhibitory effects on both non‐adaptive and adaptive immune responses. Of the non‐adaptive responses, the downregulation of the AA cascade identified in the transcriptomic data from the APOE*3Leiden.CETP mice is in line with the significant in both hepatic HETE concentrations and ALOX5AP expression (a key regulator of 5‐HETE and leukotriene B4 synthesis) observed in the AMLN *ob/ob* model. Interestingly, the enzyme HSD17B13A, for which a loss‐of‐function variant is associated with a markedly reduced risk of progression from steatosis to steatohepatitis, displays high activity towards both HETEs and leukotrienes.^39^

Hall et al demonstrated that increased inflammation, oxidative damage and liver injury in NASH are associated with pronounced changes in membrane AA remodelling, characterised by AA enrichment in pericentral hepatocytes via upregulation of LPCAT2 expression and subsequent release via PLA2 for the generation of pro‐inflammatory HETEs.^40^ Icosabutate induced the converse effect, that is, a significant decrease in LPCAT2 expression, reduced membrane AA content and lowered HETE formation in AMLN *ob/ob* mice. Icosabutate's effects on AA metabolism, not seen in response to OCA, may thus act as a key differentiator with regard to the respective effects of each drug on liver injury and inflammation. In addition to PC‐AA, the decrease in DAG‐AA after icosabutate treatment may also have relevance, as DAG is potentially a more important source of AA than membrane PC for eicosanoid synthesis in the liver.^32^ The reason for the decrease in hepatic AA stores in response to icosabutate are beyond the scope of the current work but inhibition of AA synthesis in vitro by both EPA and α‐substituted EPA has previously been reported.^41^

With respect to the improvements in hepatic oxidative stress, incorporation of highly unsaturated omega‐3 fatty acids into hepatocyte membranes could potentially increase susceptibility of hepatocytes to lipid peroxidation.^42^ However, icosabutate was able to markedly decrease both hepatic GSSG and hepatic oxPLs in AMLN *ob/ob* mice, an effect that likely benefits from the fact that α‐substituted EPA avoids incorporation into phospholipids.^41^ The decrease in PC‐AA in response to icosabutate may also contribute to the reduction in oxPL by reducing membrane susceptibility to peroxidation. Indeed, the percentage reductions in oxPL (which comprises both oxidised AA‐ and linoleic acid‐containing phospholipids) closely mirror the decreases in PC‐AA.

Interestingly, in AMLN diet‐induced NASH in Ldlr^−/−^ mice, it has recently been shown that neutralisation of oxPLs substantially reduces hepatic apoptosis,^43^ a crucial driver of liver injury in NASH.^44^ This corresponds well with our findings in AMLN *ob/ob* mice, with a decrease in oxPLs occurring in conjunction with a significant reduction in apoptosis in response to icosabutate treatment. The overall changes induced by icosabutate also correspond with the substrate‐overload liver injury model of NASH pathogenesis, where lipotoxic lipid species sequentially trigger oxidative stress, apoptosis and stellate cell activation.^1^ Furthermore, although OCA was effective in reducing steatosis, there was no effect on oxidative stress, oxPLs or apoptosis, suggesting that a quantitative reduction in liver fat alone is insufficient for reducing fibrosis in the AMLN *ob/ob* mouse model.

In relation to the effects upon atherogenic plasma lipids and hepatic lipid metabolism, the comprehensive data obtained in the APOE*3Leiden.CETP mice studies clearly demonstrate that icosabutate improves lipid and lipoprotein kinetics in conjunction with transcriptional activation of hepatic genes involved in fatty acid metabolism and handling. When combined with the biochemical measurement of plasma and liver TAG contents, the data demonstrate that TAGs are taken up by the liver and metabolically processed, resulting in a continuous removal of plasma TAGs in icosabutate‐treated animals. We also observed increased transcription of genes involved in cholesterol synthesis, likely regulated by increased SREBF2‐dependent pathway activation^45^ in response to a decrease in hepatic cholesterol in icosabutate‐treated animals. Using pathway analysis, a role for SREBF2 activation was predicted in icosabutate‐treated animals, but not by treatment with fenofibrate. This suggests that SREBF2‐mediated effects on LDL‐R expression and cholesterol synthesis are among the PPARα‐independent effects induced by icosabutate. Overall, the data suggest that the decreases in atherogenic lipids and lipoproteins previously described in the clinic are secondary to increased hepatic clearance with minimal contributions from a decrease in hepatic output and/or increased peripheral TAG lipolysis.^16^ Importantly, the effects observed in the APOE*3Leiden.CETP mice confirm that icosabutate significantly inhibits the development of atherosclerosis in association with reductions in atherogenic plasma lipids.

A limitation of the current work in relation to clinical translatability is that OCA displayed minimal effects on fibrosis in the current AMLN *ob/ob* mouse model of NASH, yet has demonstrated beneficial efficacy in humans.^21^ The reason for the lack of effect seen with OCA treatment in the current model is uncertain but as improvements in fibrosis have been observed after 16 weeks treatment with OCA in the same model,^46^ the 8 week treatment period employed in the current study may have been too short.

Another potential limitation is that although the data demonstrate reductions in lipid species that have established negative effects on NASH and CVD, potential positive contributions of icosabutate metabolites are not accounted for. Specific omega‐3 metabolites, referred to as specialised pro‐resolving mediators (SPMs), have anti‐fibrotic effects and have even been shown to reverse established fibrosis (see review by Musso et al^47^). Although the main oxygenated metabolites of icosabutate have been identified in vivo, their comparative function vs oxygenated EPA metabolites are still being established.

In summary, the data demonstrate that icosabutate, a liver‐targeted, semi‐synthetic EPA derivative, effectively targets both atherogenesis and hepatic fibrosis in association with increased clearance of atherogenic plasma lipids and decreased hepatic stellate cell activation respectively. Icosabutate may thus provide a promising and novel therapeutic approach to the dual treatment of liver‐ and CV‐related morbidity and mortality in NASH patients.

## CONFLICT OF INTEREST

Northsea Therapeutics BV acquired the commercial rights for icosabutate. JK and SF are paid consultants and have stock options in Northsea Therapeutics BV. DF and TS are employees of NorthSea Therapeutics BV but had no role in data collection. SF acts as a paid consultant for the following companies: 89 Bio, Amgen, Axcella Health, Blade Therapeutics, Bristol Myers Squibb, Can‐Fite Biopharma, ChemomAb, Escient Pharmaceuticals, Forbion, Galmed, Gordian Biotechnology, Glycotest, Glympse Bio, In sitro, Morphic Therapeutics, Novartis, Ono Pharmaceuticals, Scholar Rock, Surrozen. All other authors have no conflict of interest to declare.

## Supporting information

Fig S1

Fig S2

Fig S3

Supplementary Material
