# Supplementary figures and images for: Dual targeting of hepatic fibrosis and atherogenesis by icosabutate, an engineered eicosapentaenoic acid derivative
Source: Liver Int. 2020 Oct 28;40(11):2860–76. doi: 10.1111/liv.14643 (PMC7702170; doi:10.1111/liv.14643)

## Slide 1
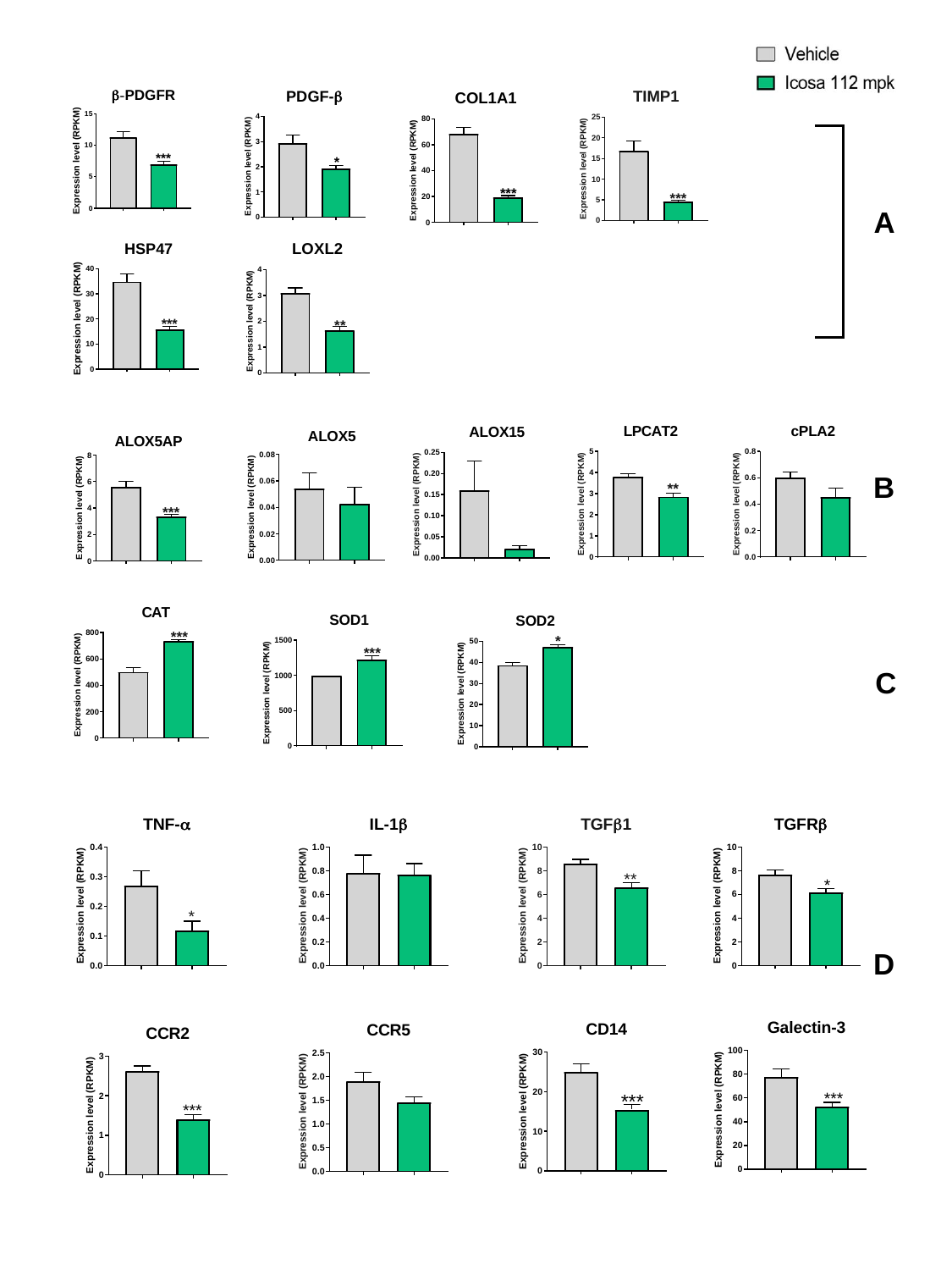

Supplement: Supplementary file 1 — Fig S1 [file LIV-40-2860-s001.pptx]

## Slide 1
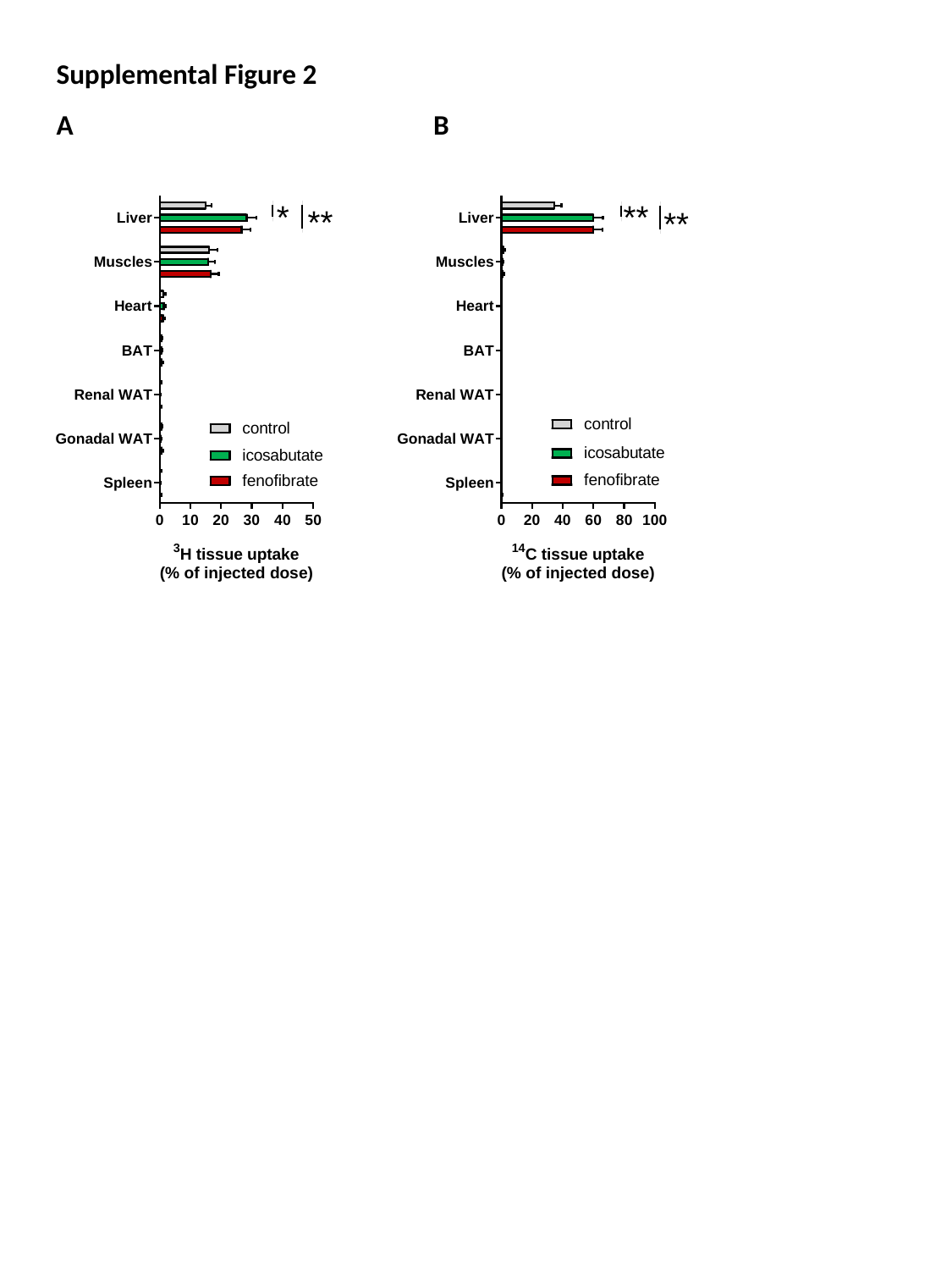

Supplemental Figure 2
A
B

Supplement: Supplementary file 2 — Fig S2 [file LIV-40-2860-s002.pptx]

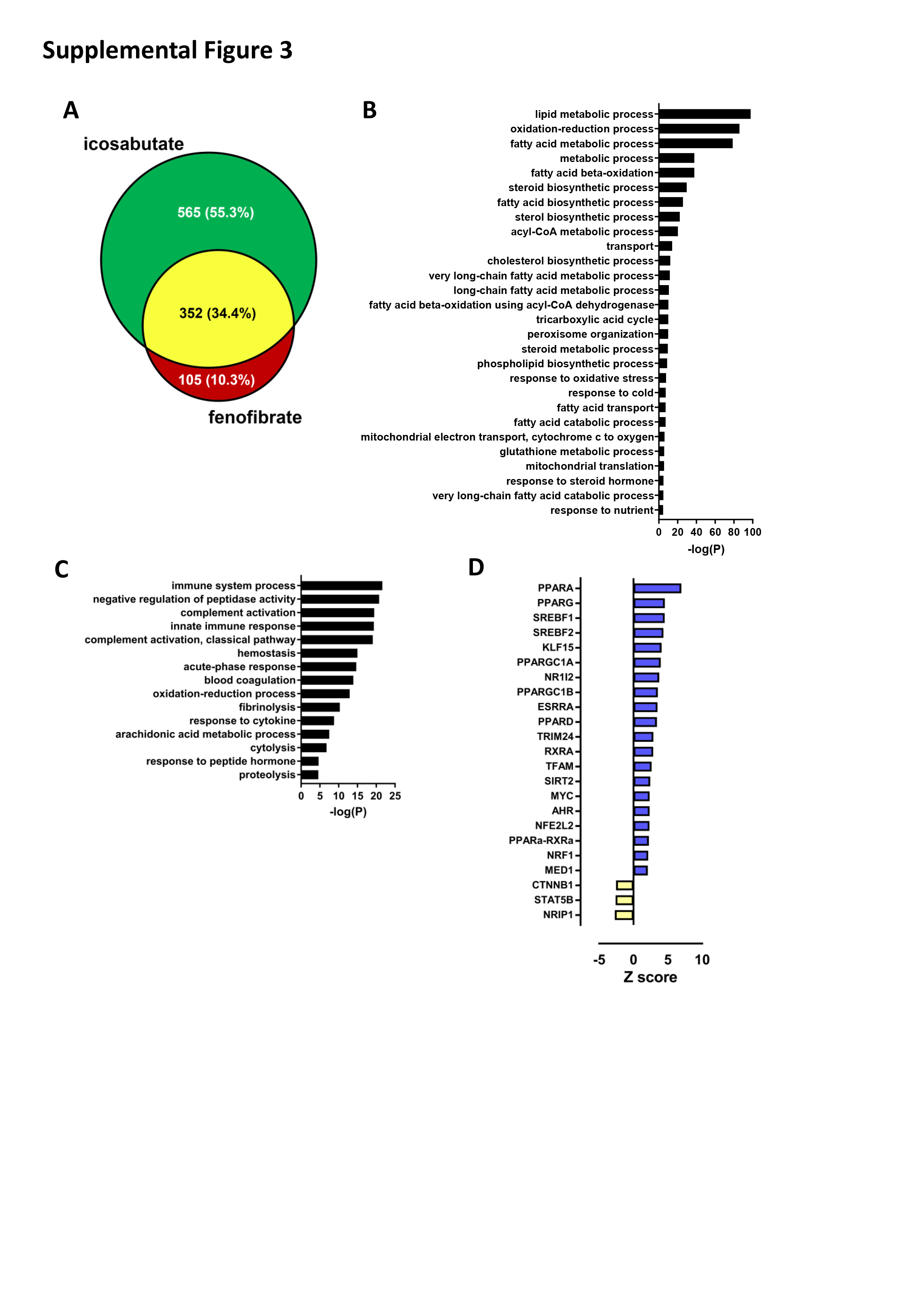

Supplement: Supplementary file 3 — Fig S3 [file LIV-40-2860-s003.tif]
