# Supplementary material for: Dual targeting of hepatic fibrosis and atherogenesis by icosabutate, an engineered eicosapentaenoic acid derivative
Source: Liver Int. 2020 Oct 28;40(11):2860–76. doi: 10.1111/liv.14643 (PMC7702170; doi:10.1111/liv.14643)
Supplement: Supplementary file 4 — Supplementary Material [file LIV-40-2860-s004.docx]

Supplementary Methods and Data

**Dual targeting of hepatic fibrosis and atherogenesis by icosabutate, an engineered eicosapentaenoic acid derivative**

Geurt Stokman, Anita M. van den Hoek, Ditte Denker Thorbekk, Elsbet J. Pieterman, Sanne Skovgård Veidal, Brittany Basta, Marta Iruarrizaga-Lejarreta, José W. van der Hoorn, Lars Verschuren, Jimmy F.P. Berbée, Patrick C.N. Rensen, Tore Skjæret, Cristina Alonso, Michael Feigh, John J.P. Kastelein, Scott L. Friedman, Hans M.G. Princen, David A. Fraser

**Analytical procedures**

***ob/ob* NASH study**

*Biochemical and histological analyses*

A terminal blood sample was collected from the tail vein in non-fasted mice and used for plasma biochemistry. Animals were sacrificed by cardiac puncture under isoflurane anaesthesia. Biochemical and histological analyses were performed as reported previously (1). Plasma analytes included alanine aminotransferase (ALT), aspartate aminotransferase (AST) and triacylglycerol (TAG). Liver homogenates were analysed for TAG. Paraformaldehyde-fixed liver pre- and post-biopsies were paraffin-embedded, sectioned, and stained with hematoxylin-eosin, laminin (Dako, Glostrup, Denmark), anti-type I collagen (Col1a1, pre and post biopsies), anti-galectin-3 (gal-3, Biolegend, San Diego, CA, United States), alpha-smooth muscle actin (α-SMA, pre and post biopsies) and anti-Desmin (pre and post biopsies) (Abcam, Cambridge, UK). Because treatment paradigms affected total liver weight, quantitative data on liver biochemistry (liver TAG, hydroxyproline (HYP)) and histology (liver lipid, Col1a1, Gal-3, α-SMA, desmin) were expressed as whole-liver amounts by multiplying individual terminal liver weight with the corresponding liver lipid concentration (biochemistry data) or percent fractional area (histology data), respectively.

Terminal deoxynucleotidyl transferase-mediated dUTP nick end-labeling (TUNEL) stains were performed in paraffin-embedded liver biopsies using a TMR red in situ cell death detection kit (Roche, Indianapolis, IN) according to manufacturer’s instructions, using previously described methods (2). 4’,6-Diamidino-2-phenylindole (DAPI) was used as a cell nuclear counterstain.

*Transcriptome analysis*

Hepatic transcriptome analysis was performed by RNA sequencing on RNA extracts from terminal liver samples (15 mg fresh tissue), as described in detail elsewhere (1). The RNA quantity was measured using NanoDrop™ 2000 (Thermo Scientific, Eugene, OR). The RNA quality was determined using a bioanalyzer with RNA 6000 Nano kit (Agilent, Waldbronn, Germany). RNA sequence libraries were prepared from 100ng total RNA using NEBNext® Ultra™ II Directional RNA Library Prep Kit for Illumina® (New England Biolabs, MA, United States) and sequenced on the NextSeq 500 (Illumina, San Diego, CA, United States) with NSQ 500 hi-Output KT v2 (75 CYS, Illumina, San Diego, CA, United States). Reads were aligned to the GRCm38 v89 Ensembl Mus musculus genome using STAR v.2.5.2a with default parameters (4). Genes with a Benjamini and Hochberg adjusted P ≤ 0.05 (5% False Discovery Rate) were regarded as statistically significantly regulated.

***Hepatic lipidomic analyses***

For detailed lipidomics, liver metabolic profiles were semi-quantified as described previously (5). Two separate ultra-high-performance liquid chromatography (UHPLC)-Time of Flight-MS based platforms analyzing methanol and chloroform/methanol extracts were used. Identified ion features in the methanol extract platform included bile acids and oxidised fatty acids. The chloroform / methanol extract platform provided coverage over glycerolipids, cholesterol esters, sphingolipids, diacylglycerophospholipids, and acyl-ether-glycerophospholipids. Lipid nomenclature and classification follows the LIPID MAPS convention, [www.lipidmaps.org](http://www.lipidmaps.org). A specific metabolite extraction procedure was performed for each platform. Metabolite extraction procedures, chromatographic separation conditions and mass spectrometric detection conditions are also detailed in (6, 7). Metabolomics data were pre-processed using the TargetLynx application manager for MassLynx 4.1 (Waters Corp., Milford, MA). Intra-batch (multiple internal standard response correction) and inter-batch (variable specific inter-batch single point external calibration using repeat extracts of a commercial serum sample) normalization followed the procedure described in (6). Metabolomic data are represented as means ± SEM. Differences between groups were tested using Student’s t-test. Significance was defined as P<0.05. All calculations were performed using statistical software package R v.3.1.1 (R Development Core Team, 2011; <https://cran.r-project.org/>). Two outliers were removed (one from the OCA group and one from the icosabutate 135mpk group) as identified based on their appearance outside the Hotelling’s T2 ellipse with confirmation by Chauvenet’s criterion.

Extraction Method and UPLC-MS conditions for assessment of hepatic reduced glutathione (GSH) and oxidized glutathione (GSSG) was performed as described previously (8).

***LX-2 proliferation assay***

LX-2 cells (supplied by Scott Friedman, Mount Sinai School of Medicine, New York) were plated at 5000 cells per well in 96 well plates. After overnight serum starvation in DMEM supplemented with 0.1% BSA (without antibiotic), cells were incubated in medium (containing 0.6mM BSA) with either 10, 25, 50 & 75 μM icosabutate or 75 μM oleic acid for a further 24 hours. DMSO was used as vehicle control in parallel at each time point. After 24 hours of fatty-acid exposure the cells were labelled with BrdU for 2 hours at 37°C in a 5% CO2 incubator. We used the Cell Proliferation ELISA, BrdU colorimetric kit (Roche, NY) and followed the manufacturer’s protocol. Absorbance was measured at 370 nm with reference wavelength at 492 nm. To measure cytotoxicity, cell cultures were prepared and treated with compounds as for the LX-2 cell proliferation assay. At 24 hours, MTS assays were performed using CellTiter 96 AQueous One Solution Cell Proliferation Assay kit (Promega, WI) according to the manufacturer’s protocol after 2 hours of incubation at 37°C in a 5% CO2 incubator.

**APOE*3Leiden.CETP study**

*Analysis of plasma lipid concentration and composition in mouse samples*

Animals were sacrificed by CO_2_ asphyxiation. Liver, heart tissue and aortic roots were fixed in formalin or snap-frozen in liquid nitrogen. Plasma TC and TAG levels were determined by CHOD-PAP and GPO-PAP, respectively, from Roche Diagnostics (Almere, The Netherlands) according to the supplied protocol. For plasma lipid and lipoprotein metabolism studies blood samples and VLDL particle clearance and production were determined as described below at week 4. For the atherosclerosis study blood samples were obtained every 2 to 3 weeks after 4 hours fasting, transferred to EDTA collection tubes and plasma was collected. Total cholesterol exposure was expressed as mM cholesterol times the duration of the study. HDL-C was measured in plasma supernatants after precipitation of ApoB-containing particles as described earlier (9); non-HDL-C was calculated by subtracting HDL-C levels from total cholesterol levels.

Plasma lipoprotein profiles were determined by fast-performance liquid chromatography (FPLC) using an AKTA apparatus (Amersham Biosciences, Little Chalfont, UK) followed by fractional cholesterol or triglyceride measurement as described above. Fecal bile acids and neutral sterols were determined by gas chromatography as described previously (10, 11).

*Liver lipid contents and enzyme activity assays*

Liver lipids were determined as described previously (12). Approximately 50 mg of tissue was homogenized in phosphate buffered saline. Lipids were extracted and separated by high performance thin layer chromatography on silica gel plates. Lipid spots were stained with color reagent (5 g MnCl_2_·4H_2_O, 32 mL 95-97% H_2_SO_4_ added to 960 mL of CH_3_OH:H_2_O 1:1 [v/v]) and corrected for total protein content. AST and ALT levels were measured in plasma using a spectrophotometric activity assay (Reflotron Plus system, Roche).

Post-heparin plasma from 4-hour fasted mice was collected from the tail vein at 20 minutes after intraperitoneal injection of heparin (0.5 IU/g body weight). Post-heparin plasma triacylglycerol hydrolase activity was determined in the presence or absence of 1 M NaCl to estimate both the hepatic lipase and lipoprotein lipase activity. Lipoprotein lipase activity was calculated as the portion of total lipase activity inhibited by 1 M NaCl (11).

*Production and clearance of VLDL particles*

To study VLDL production and clearance, 4-hour fasted mice were anesthetised using acepromazine (Ventranquil), midazolam (Dormicum) and fentanyl. For VLDL production, a tail vein injection of Tyloxapol (Sigma Aldrich) (500 mg/kg body weight) was given at t=0 minutes and blood samples were collected directly or at 15, 30, 60 and 90 minutes followed by isolation of VLDL particles using density gradient ultracentrifugation (9, 13).

To study VLDL clearance mice received a tail vein injection containing 80 nm-sized VLDL-like emulsion particles labeled with glycerol tri[^3^H]oleate (triolein) and [^14^C]-cholesteryl oleate (both from GE Healthcare, Little Chalfont, UK) (9, 13) at a dose of 1 mg TG per mouse. Blood samples were collected at 2, 5, 10, 20 and 30 minutes. Liver, heart, spleen, hind leg muscle (*biceps femoris anterior*), and perigonadal, retroperitoneal (renal), and brown adipose tissue were collected for label quantification.

*RNA isolation and transcriptomics*

For transcriptome analysis, mRNA was isolated from liver tissue using RNA-Bee (Bio-Connect, Huissen, The Netherlands) and purified using NucleoSpin RNA Clean-up (Macherey-Nagel, Düren, Germany). The integrity of each RNA sample was examined by Agilent Lab-on-a-chip technology using the RNA 6000 Nano LabChip kit and a bioanalyzer 2100 (both Agilent Technologies, Amstelveen, The Netherlands). The Illumina® TotalPrep™ RNA Amplification Kit (Ambion, art.No.AM-IL1791) was used to synthesize biotin labeled cRNA starting with 500 ng total RNA. The concentration of the labeled cRNA was measured using the Nanodrop spectrophotometer. The amount of biotinylated cRNA which was hybridized onto the MouseRef-8 Expression BeadChip was 750 ng. Illumina’s Genomestudio v1.1.1 software with the default settings advised by Illumina was used for Gene Expression analysis. All the quality control data of this BeadChip were within specifications of the microarray service provider (Service XS, Leiden, the Netherlands). Gene expression data analysis and preprocessing was performed using ArrayAnalysis (URL: http://www.arrayanalysis.org/). This web-based tool generates probe-level background subtracted expression values which were used as input for lumi package (14) of the R/Bioconductor (URL: http://www.bioconductor.org; http://www.r-project.org) to perform quality control and a quantile normalization. Pathway enrichment and upstream regulator analysis were performed using Ingenuity Pathway Analysis (IPA) software (Ingenuity Systems Inc., Redwood City, CA) as described earlier (12, 15). The activation state of transcription factors was determined based on the observed differential gene expression and used to calculate an activation Z score for each transcription factor.

*Histochemistry and atherosclerotic plaque analysis*

Formalin fixed aortic roots were embedded in paraffin in a routine fashion and 5 μm thick sections were cut and mounted on 3-aminopropyltriethoxysilane coated glass slides. After dewaxing in xylene and an ethanol gradient, slides were stained with hematoxylin, 0.25% (w/v) phloxine and counterstained with 0.25% (w/v) saffron in ethanol. Digital images of 4 serial cross-sections were collected at 50μm intervals using an Olympus BX51 microscope (Olympus, Hamburg, Germany). Morphometric analysis of lesion area was performed using cell^D software v2.7 (Olympus Soft Imaging Solutions, Hamburg, Germany) and lesion severity was scored in a blinded fashion according to the classification by the American Heart Association, as described previously (16).

*Protein expression assays*

LDL-R and LRP expression was determined in snap-frozen liver tissue (17). In short, tissue slices were lysed in 50 mM Tris-HCl (pH 7.4), 150 mM NaCl, 0.25% deoxycholic acid, 1% NP-40 and 1 mM EDTA supplemented with Complete Protease Inhibitor Cocktail (Roche Diagnostics, Almere, The Netherlands), 1 mM phenylmethylsulfonyl fluoride and 1 mM Na_3_VO_4_. Protein samples were heat-denatured in Laemmli buffer (Sigma Aldrich), separated by SDS-PAGE followed by blotting on polyvinylidene difluoride membranes. Immunoblots were labeled with goat-anti-mouse LDL-R (R&D Systems) and mouse-anti-tubulin (Sigma Aldrich) antibodies, followed by labeling with relevant HRP-conjugated secondary antibodies (Cell Signaling, Leiden, The Netherlands). Immunoblots were developed using West Femto Super Signal ECL (Thermo Fisher Scientific) and imaged on a Chemi Doc-it system (Thermo Fisher Scientific). Densitometric analysis was performed using ImageJ software.

**References**

1. Kristiansen MN, Veidal SS, Rigbolt KT, Tolbol KS, Roth JD, Jelsing J, et al. Obese diet-induced mouse models of nonalcoholic steatohepatitis-tracking disease by liver biopsy. World J Hepatol 2016;8:673-684.

2. Jackson AC, Kammouni W, Zherebitskaya E, Fernyhough P. Role of oxidative stress in rabies virus infection of adult mouse dorsal root ganglion neurons. J Virol 2010;84:4697-4705.

3. Kleiner DE, Brunt EM, Van Natta M, Behling C, Contos MJ, Cummings OW, et al. Design and validation of a histological scoring system for nonalcoholic fatty liver disease. Hepatology 2005;41:1313-1321.

4. Dobin A, Davis CA, Schlesinger F, Drenkow J, Zaleski C, Jha S, et al. STAR: ultrafast universal RNA-seq aligner. Bioinformatics 2013;29:15-21.

5. Barr J, Caballeria J, Martinez-Arranz I, Dominguez-Diez A, Alonso C, Muntane J, et al. Obesity-dependent metabolic signatures associated with nonalcoholic fatty liver disease progression. J Proteome Res 2012;11:2521-2532.

6. Martinez-Arranz I, Mayo R, Perez-Cormenzana M, Minchole I, Salazar L, Alonso C, et al. Enhancing metabolomics research through data mining. J Proteomics 2015;127:275-288.

7. Alonso C, Fernandez-Ramos D, Varela-Rey M, Martinez-Arranz I, Navasa N, Van Liempd SM, et al. Metabolomic Identification of Subtypes of Nonalcoholic Steatohepatitis. Gastroenterology 2017;152:1449-1461 e1447.

8. van den Hoek AM, Pieterman EJ, van der Hoorn JW, Iruarrizaga-Lejarreta M, Alonso C, Verschuren L, et al. Icosabutate Exerts Beneficial Effects Upon Insulin Sensitivity, Hepatic Inflammation, Lipotoxicity, and Fibrosis in Mice. Hepatol Commun 2020;4:193-207.

9. van der Tuin SJ, Kuhnast S, Berbee JF, Verschuren L, Pieterman EJ, Havekes LM, et al. Anacetrapib reduces (V)LDL cholesterol by inhibition of CETP activity and reduction of plasma PCSK9. J Lipid Res 2015;56:2085-2093.

10. Post SM, Duez H, Gervois PP, Staels B, Kuipers F, Princen HM. Fibrates suppress bile acid synthesis via peroxisome proliferator-activated receptor-alpha-mediated downregulation of cholesterol 7alpha-hydroxylase and sterol 27-hydroxylase expression. Arterioscler Thromb Vasc Biol 2001;21:1840-1845.

11. Post SM, de Crom R, van Haperen R, van Tol A, Princen HM. Increased fecal bile acid excretion in transgenic mice with elevated expression of human phospholipid transfer protein. Arterioscler Thromb Vasc Biol 2003;23:892-897.

12. Bijland S, Pieterman EJ, Maas AC, van der Hoorn JW, van Erk MJ, van Klinken JB, et al. Fenofibrate increases very low density lipoprotein triglyceride production despite reducing plasma triglyceride levels in APOE*3-Leiden.CETP mice. J Biol Chem 2010;285:25168-25175.

13. Geerling JJ, Boon MR, van der Zon GC, van den Berg SA, van den Hoek AM, Lombes M, et al. Metformin lowers plasma triglycerides by promoting VLDL-triglyceride clearance by brown adipose tissue in mice. Diabetes 2014;63:880-891.

14. Du P, Kibbe WA, Lin SM. lumi: a pipeline for processing Illumina microarray. Bioinformatics 2008;24:1547-1548.

15. Pouwer MG, Pieterman EJ, Verschuren L, Caspers MPM, Kluft C, Garcia RA, et al. The BCR-ABL1 Inhibitors Imatinib and Ponatinib Decrease Plasma Cholesterol and Atherosclerosis, and Nilotinib and Ponatinib Activate Coagulation in a Translational Mouse Model. Front Cardiovasc Med 2018;5:55.

16. Delsing DJ, Offerman EH, van Duyvenvoorde W, van Der Boom H, de Wit EC, Gijbels MJ, et al. Acyl-CoA:cholesterol acyltransferase inhibitor avasimibe reduces atherosclerosis in addition to its cholesterol-lowering effect in ApoE*3-Leiden mice. Circulation 2001;103:1778-1786.

17. Kuhnast S, van der Hoorn JW, Pieterman EJ, van den Hoek AM, Sasiela WJ, Gusarova V, et al. Alirocumab inhibits atherosclerosis, improves the plaque morphology, and enhances the effects of a statin. J Lipid Res 2014;55:2103-2112.

**Supplementary Figures.**

**Supplemental Figure 1.** Effects of 4 weeks treatment with icosabutate (112mpk) on hepatic mRNA transcripts regulating (Section A) fibrosis and fibrolysis, (Section B) phospholipid/arachidonic metabolism, (Section C) enzymatic antioxidants and (Section D) inflammation in AMLN fed *ob/ob* mice. Data are presented as mean expression levels (RPKM) ± SEM, * p<0.05, ** p<0.01, *** p<0.001 *vs.* vehicle.

**Supplemental Figure 2.** Tissue uptake of (A) glycerol tri[^3^H]oleate-derived activity and (B) [^14^C]cholesteryl oleate-derived activity expressed as percentage of injected dose. BAT, brown adipose tissue; WAT, white adipose tissue. Effects of treatments on hepatic lipid concentrations (C) and faecal bile acids and neutral sterols (D). Data are presented as mean ± SEM, **P*≤0.05, ***P*≤0.01, ****P*≤0.001

**Supplemental Figure 3.** Liver tissue was collected from male APOE*3Leiden.CETP mice that were fed WTD and received vehicle, 112 mpk icosabutate or 30 mpk fenofibrate for 4 weeks. (A) The total number of differentially expressed genes (DEG) for each treatment compared to the control group and overlap between treatments is shown in a Venn diagram. Enrichment analysis of (B) upregulated and (C) downregulated pathways in response to icosabutate treatment. (D) Predicted activation of upstream pathway regulators based on differentially transcribed genes in the icosabutate group (*P*≤0.05 versus control group). Regulators are ranked based on Z score. Scores of ≤ -2 (inhibition) and ≥ 2 (activation) are shown as yellow and blue bars, respectively.
